# Supplementary material for: Assessment of the Effects of Physiotherapy on Back Care and Prevention of Non-Specific Low Back Pain in Children and Adolescents: A Systematic Review and Meta-Analysis
Source: Healthcare (Basel). 2024 May 16;12(10):1036. doi: 10.3390/healthcare12101036 (PMC11121698; doi:10.3390/healthcare12101036)
Supplement: Supplementary file 1 [file healthcare-12-01036-s001.zip › healthcare-2977919-supplementary.pdf]

Supplementary Table S1. PRISMA Checklist.

Section and Topic	Item #	Checklist item	Location where item is reported
**TITLE**			
Title	1	Identify the report as a systematic review.	Page 1
**ABSTRACT**			
Abstract	2	See the PRISMA 2020 for Abstracts checklist.	Page 1
**INTRODUCTION**			
Rationale	3	Describe the rationale for the review in the context of existing knowledge.	Page 2
Objectives	4	Provide an explicit statement of the objective(s) or question(s) the review addresses.	Page 2
**METHODS**			
Eligibility criteria	5	Specify the inclusion and exclusion criteria for the review and how studies were grouped for the syntheses.	Page 2
Information sources	6	Specify all databases, registers, websites, organisations, reference lists and other sources searched or consulted to identify studies. Specify the date when each source was last searched or consulted.	Page 2
Search strategy	7	Present the full search strategies for all databases, registers and websites, including any filters and limits used.	Supplementary Table S2
Selection process	8	Specify the methods used to decide whether a study met the inclusion criteria of the review, including how many reviewers screened each record and each report retrieved, whether they worked independently, and if applicable, details of automation tools used in the process.	Page 3
Data collection process	9	Specify the methods used to collect data from reports, including how many reviewers collected data from each report, whether they worked independently, any processes for obtaining or confirming data from study investigators, and if applicable, details of automation tools used in the process.	Page 3
Data items	10a	List and define all outcomes for which data were sought. Specify whether all results that were compatible with each outcome domain in each study were sought (e.g. for all measures, time points, analyses), and if not, the methods used to decide which results to collect.	Page 3
	10b	List and define all other variables for which data were sought (e.g. participant and intervention characteristics, funding sources). Describe any assumptions made about any missing or unclear information.	Supplementary Table S3
Study risk of bias assessment	11	Specify the methods used to assess risk of bias in the included studies, including details of the tool(s) used, how many reviewers assessed each study and whether they worked independently, and if applicable, details of automation tools used in the process.	Page 3
Effect measures	12	Specify for each outcome the effect measure(s) (e.g. risk ratio, mean difference) used in the synthesis or presentation of results.	Page 3
Synthesis methods	13a	Describe the processes used to decide which studies were eligible for each synthesis (e.g. tabulating the study intervention characteristics and comparing against the planned groups for each synthesis (item #5)).	Page 3

Section and Topic	Item #	Checklist item	Location where item is reported
	13b	Describe any methods required to prepare the data for presentation or synthesis, such as handling of missing summary statistics, or data conversions.	Page 3
	13c	Describe any methods used to tabulate or visually display results of individual studies and syntheses.	Page 3
	13d	Describe any methods used to synthesize results and provide a rationale for the choice(s). If meta-analysis was performed, describe the model(s), method(s) to identify the presence and extent of statistical heterogeneity, and software package(s) used.	Page 3
	13e	Describe any methods used to explore possible causes of heterogeneity among study results (e.g. subgroup analysis, meta-regression).	Page 3
	13f	Describe any sensitivity analyses conducted to assess robustness of the synthesized results.	Page 3
Reporting bias assessment	14	Describe any methods used to assess risk of bias due to missing results in a synthesis (arising from reporting biases).	Page 3
Certainty assessment	15	Describe any methods used to assess certainty (or confidence) in the body of evidence for an outcome.	Page 3 and supplementary Table S4
**RESULTS**			
Study selection	16a	Describe the results of the search and selection process, from the number of records identified in the search to the number of studies included in the review, ideally using a flow diagram.	Page 4
	16b	Cite studies that might appear to meet the inclusion criteria, but which were excluded, and explain why they were excluded.	Page 4
Study characteristics	17	Cite each included study and present its characteristics.	Page 4, Table 1
Risk of bias in studies	18	Present assessments of risk of bias for each included study.	Page 19, Table 2
Results of individual studies	19	For all outcomes, present, for each study: (a) summary statistics for each group (where appropriate) and (b) an effect estimate and its precision (e.g. confidence/credible interval), ideally using structured tables or plots.	Pages 19-26, figures 2,4
Results of syntheses	20a	For each synthesis, briefly summarise the characteristics and risk of bias among contributing studies.	Pages 19-26
	20b	Present results of all statistical syntheses conducted. If meta-analysis was done, present for each the summary estimate and its precision (e.g. confidence/credible interval) and measures of statistical heterogeneity. If comparing groups, describe the direction of the effect.	Pages 19-26
	20c	Present results of all investigations of possible causes of heterogeneity among study results.	Pages 19-26
	20d	Present results of all sensitivity analyses conducted to assess the robustness of the synthesized results.	Pages 19-26
Reporting biases	21	Present assessments of risk of bias due to missing results (arising from reporting biases) for each synthesis assessed.	Page 19, Table 2

Section and Topic	Item #	Checklist item	Location where item is reported
Certainty of evidence	22	Present assessments of certainty (or confidence) in the body of evidence for each outcome assessed.	Page 25 and supplementary Table S4
**DISCUSSION**			
Discussion	23a	Provide a general interpretation of the results in the context of other evidence.	Pages 26,27
	23b	Discuss any limitations of the evidence included in the review.	Page 28
	23c	Discuss any limitations of the review processes used.	Page 28
	23d	Discuss implications of the results for practice, policy, and future research.	Page 26
**OTHER INFORMATION**			
Registration and protocol	24a	Provide registration information for the review, including register name and registration number, or state that the review was not registered.	Page 2
	24b	Indicate where the review protocol can be accessed, or state that a protocol was not prepared.	Page 2
	24c	Describe and explain any amendments to information provided at registration or in the protocol.	Page 2
Support	25	Describe sources of financial or non-financial support for the review, and the role of the funders or sponsors in the review.	Page 28
Competing interests	26	Declare any competing interests of review authors.	Page 28
Availability of data, code and other materials	27	Report which of the following are publicly available and where they can be found: template data collection forms; data extracted from included studies; data used for all analyses; analytic code; any other materials used in the review.	NA

Supplementary Table S2

Search strategy for Meta-analysis 1

Results of the search from some of the databases consulted

Databases	Key words	Results
Cochrane Library	PHASE 1: (1) childhood; (2) adolescence; (3) children; (4) #1 OR #2 OR #3.<br>PHASE 2: (1) treatment; (2) prevention; (3) education; (4) “postural hygiene program”; (5) “physical education”; (6) “back education” ; (7) “Posture education”; (8) “Back function”; (9) physiotherapy; (10) ergonomics; (11) ”physical therapy”; (12) “exercise therapy” ; (13) “back care”; (14): #1 OR #2 OR #3 OR #4 OR #5 OR #6 OR #7 OR #8 OR #9 OR #10 OR #11 OR #12 OR #13.<br>PHASE 3: (1) “back pain”; (12) “low back pain” #1 OR #2.<br>TOTAL PHASES: 1 AND 2 AND 3.	152
Medline	PHASE 1: (1) Child*; (2) Adolescen*; (3) #1 OR #2<br>PHASE 2: (1) treatment; (2) prevention; (3) education; (4) “postural hygiene program”; (5) “physical education”; (6) “back education” ; (7) “Posture education”; (8) “Back function” ; (9) physiotherapy; (10) ergonomics; (11) ”physical therapy”; (12) “exercise therapy” (13) promotion; (14) behaviour; (15) medical attention; (16) posture; (17): #1 OR #2 OR #3 OR #4 OR #5 OR #6 OR #7 OR #8 OR #9 OR #10 OR #11 OR #12 OR #13 OR #14 OR #15 OR #16 OR #17.<br>PHASE 3: (1) “back pain”; (2)”low back pain”; (3) “back care”.<br>TOTAL PHASES: 1 AND 2 AND 3	259
Web of Science	PHASE 1: (1) Child*; (2) Adolescen*; (3) #1 OR #2<br>PHASE 2: (1) treatment; (2) prevention; (3) education; (4) “postural hygiene program” (5) “physical education”; (6) “back education” ; (7) “Posture education”; (8) “Back function” ; (9) physiotherapy; (10) ergonomics; (11) ”physical therapy”; (12) “exercise therapy” (13) promotion; (14) behaviour; (15) medical attention; (16) posture; (17): #1	431

OR #2 OR #3 OR #4 OR #5 OR #6 OR #7 OR #8 OR #9 OR #10 OR #11 OR #12 OR #13 OR #14 OR #15 OR #16 OR #17.

PHASE 3: (1) “back pain”; (2)”low back pain”; (3) “back care”.

TOTAL PHASES: 1 AND 2 AND 3

Search strategy for Meta-analysis 2

Database	Search strategy
Medline	(adolescent* OR child* OR young* OR school*) AND (“back pain” OR “low back pain” OR “back complaint” OR “back care”) AND (prevention OR education OR “postural hygiene” OR “physical education” OR “back education” OR “posture education” OR “back function” OR physiotherapy OR backpack OR ergonomics OR “physical therapy” OR “exercise therapy” OR promotion OR knowledge OR behaviour OR “cognitive behavioral therapy”)<br><br>Filters: publication date from 2012/05/01; age 0-18
Web of Science	(adolescent* OR child* OR young* OR school*) AND (“back pain” OR “low back pain” OR “back complaint” OR “back care”) AND (prevention OR education OR “postural hygiene” OR “physical education” OR “back education” OR “posture education” OR “back function” OR physiotherapy OR backpack OR ergonomics OR “physical therapy” OR “exercise therapy” OR promotion OR knowledge OR behaviour OR “cognitive behavioral therapy”)<br><br>Filters: publication date from 2012; Title/abstract
Cochrane Library	(adolescent* OR child* OR young* OR school*) AND (“back pain” OR “low back pain” OR “back complaint” OR “back care”) AND (prevention OR education OR “postural hygiene” OR “physical education” OR “back education” OR “posture education” OR “back function” OR physiotherapy OR backpack OR ergonomics OR “physical therapy” OR “exercise therapy” OR promotion OR knowledge OR behaviour OR “cognitive behavioral therapy”)<br><br>Filters: publication date from 2012/05/01
PEDro	Adolescent* “low back pain”<br><br>Filters: publication date from 2012/05/01; Match all search items (AND)
PsycInfo	(adolescent* OR child* OR young* OR school*) AND (“back pain” OR “low back pain” OR “back complaint” OR “back care”) AND (prevention OR education OR “postural hygiene” OR “physical education” OR “back education” OR “posture education” OR “back function” OR physiotherapy OR backpack OR ergonomics OR “physical therapy” OR “exercise therapy” OR promotion OR knowledge OR behaviour OR “cognitive behavioral therapy”)<br><br>Filters: publication date from 2012; Chilhood (birth-12 years), Adolescent (13-17 years)
LILACS	Adolescent* “low back pain”<br><br>Filters: publication date from 2012 to 2020
IBECS	Adolescent low back pain<br><br>Filters: match all the search items (AND)

Supplementary Table S3. Coded variables.

Type of variables	Coded variables
**Context**	(a) the country and (b) the place where the intervention was carried out (university, clinic, health center/day center, hospital, school, sports center, mixed).
**Treatment**	(a) the type of preventive physiotherapy treatment (postural hygiene, physiotherapy exercise, physical activity, others); (b) the acquisition mode of postural hygiene (acquisition of knowledge, posture training habits, body awareness training, others); (c) the teaching method of postural hygiene (theoretical, practical); (d) the type of physiotherapy exercise (stretching, strengthening, pelvic tilt exercises, breathing, posture correction, balance exercises, others, It was carried out by a specific method); (e) the type of physical activity (sports, games, others); (f) the duration of the treatment (in weeks); (g) the intensity of the treatment (number of weekly hours of treatment received by each subject); (h) the magnitude of the treatment (total number of hours received by each subject); (i) the existence of an established number of sessions; (j) the homogeneity of the treatment (whether all patients received the treatment in the same conditions); (k) the inclusion of homework; (l) the use of external agents to the therapeutic group (subjects that are not part of the therapy group, who are not professionals, but who have an influence, being able to support the subjects in attaining their therapy goals); (m) the presence of family members who act as co-therapists that continue or carry out preventive treatment at home); (n) the presence of teachers who act as co-therapists that continue or carry out preventive treatment at home; (o) the mode of application of the intervention (direct, indirect or mixed); (p) the mode of training (group, individual or mixed); (q) the use of informed consent. Regarding the characteristics of the therapists the following variables were coded: (r) the number of therapists; (s) whether or not the authors agree with the therapists; (t) the training of the therapist (physiotherapist, other); (u) the experience of the therapists (large, medium, low, mixed), and (v) the gender of therapists (men, women, mixed).
**Participants**	(a) the mean age of the subjects and the standard deviation (SD) (in years); (b) the gender of the sample (percentage of males); (c) the physical activity level of subjects during the intervention (low, moderate, regular), and (d) whether or not they had undertaken previous treatments, (e) the percentage of participants with low back pain, and (f) the percentage of participants with spinal deformity.
**Methodology**	(a) how the subjects were allocated to the treatments (randomly vs. nonrandomly); (b) the type of control group (nonactive vs. active); (c) the sample size; (d) the attrition in the posttest; (e) the attrition difference in the posttest.
**Extrensic**	(a) the year of the study; (b) the profession of the first author (physiotherapist, ergonomist, teacher, physician, other), (c) the publication of the study (published vs. un published) and, (d) the publication source (paper, book chapter, book or monograph, unpublished manuscript, congress communication, technical report, doctoral thesis, and others).

Supplementary Table S4. GRADE system.

Author(s): José Manuel García Moreno, Inmaculada Calvo Muñoz, Antonia Gómez Conesa, José Antonio López López

Question: Preventive physiotherapy any treatment compared to control for back care in children and adolescents

Setting:

Bibliography:

Certainty assessment							№ of patients		Effect		Certainty	Importance
№ of studies	Study design	Risk of bias	Inconsistency	Indirectness	Imprecision	Other considerations	Preventive physiotherapyany treatment	control	Relative (95% CI)	Absolute (95% CI)		
**Back care related behaviour (assessed with: schoolbag weight, behaviour in daily life test, behaviour in a trial test)**												
19	randomised trials	serious [a]	not serious	serious [b]	not serious	very strong effect size	2226	1930	-	SMD **1.48 SD higher** (0.40 higher to 2.56 higher)	⨁⨁⨁◯ MODERATE	CRITICAL
**Back care related knowledge (assessed with: knowledge questionnaires)**												
17	randomised trials	serious [a]	not serious	serious [b]	not serious	very strong effect size	2387	2032	-	SMD **1.41 SD higher** (1.05 higher to 1.76 higher)	⨁⨁⨁◯ MODERATE	CRITICAL

CI: Confidence interval; **SMD:** Standardised mean difference

Explanations

a. Some studies had high risk of bias

b. Different tools are used to measure this variable, so there is no homogeneity in this section.
